# Supplementary material for: Quantifying ethnic segregation in cities through random walks
Source: Nat Commun. 2022 Oct 3;13:5809. doi: 10.1038/s41467-022-33344-3 (PMC9530170; doi:10.1038/s41467-022-33344-3)
Supplement: Supplementary file 1 — Supplementary Information [file 41467_2022_33344_MOESM1_ESM.pdf]

Supplementary Information to "Quantifying ethnic segregation in cities through random walks"

Sandro Sousa[1] and Vincenzo Nicosia[1]

[1]*School of Mathematical Sciences, Queen Mary University of London, London E1 4NS, United Kingdom*

Supplementary Note 1. CORRELATIONS AT MULTIPLE SPATIAL SCALES

In addition to the class coverage time analysis in the main manuscript, we look at correlations among the nodes of G at different spatial scales. We adopted the multifractal detrended fluctuation analysis (DFA), a non-linear method which allows to detect the presence of long-term correlations of a time series [1]. With a a slightly different set-up, random walks are defined on the graph G representing the UK cities at three spatial scales (geographic delineations), the Wards level, Lower Layer Super Output Areas (LSOA) and Output Areas (OA) respectively. We set φ_i equal to the Shannon entropy of the ethnicity distribution at node i. In particular, for every node i in G, we call p_i^γ the number of people of ethnic group γ living at i (as obtained from the UK Census data, with $\Gamma = 250$ different ethnicities) and we define:

$$p_i = p_i^\gamma = \frac{x_i}{\sum_\gamma x_i^\gamma}. \tag{1}$$

Hence, the node population entropy is given by:

$$\varphi_i = \sum_{\gamma=1}^{C} p_i^\gamma \log\left[p_i^\gamma\right]. \tag{2}$$

A random walk on G produces the time series $y_i(t) = \{\varphi_{i0}, \varphi_{i1}, \varphi_{i2}, ...\}$ of the population entropy of the nodes traversed. The length T of this time-series is fixed and defined according to the number of nodes in G and the spatial scale. For the detrended non fluctuation analysis [1], we compute the profile of $y_i(t)$, obtained by subtracting the mean value $\langle y_i \rangle$ of the time series:

$$X_t \equiv \sum_{i=1}^{t} (Y_i - \langle Y \rangle), \quad t = 1, ..., T \tag{3}$$

Next, the profile X_t is divided in non-overlapping segments of equal length ε and the local linear trend is calculated by a least-square linear fit of each segment. Let K_t be the resulting piece of this process, then, the root-mean-square fluctuation of this detrended time series is calculated by:

$$F(\varepsilon) = \sqrt{\frac{1}{T}\sum_{t=1}^{T}(X_t - K_t)^2} \tag{4}$$

The local variance $\sigma^2(\ell, \varepsilon)$ is obtained and the structure function $F(\varepsilon)$ is evaluated by averaging $\sigma^2(\ell, \varepsilon)$ over all time windows whose length equals ε and $F(\varepsilon)$ is plotted as a function of ε.

If the probability to find the edge (i, j) connecting node i to node j does not depend on the values φ_i and φ_j, then the fluctuations of the corresponding time series $Y_i(t)$ obtained from a random walk will be indistinguishable from an uncorrelated Gaussian noise $F(\varepsilon) \approx \varepsilon^{1/2}$. On the other hand, $F(\varepsilon) \approx \varepsilon^\alpha$ with $\alpha \neq 1/2$ signals the presence of φ-correlations. For all UK cities in Fig. 9, φ-correlations can be observed in the regime below the transition point, and the scaling exponent α shows the magnitude of such correlation. Above the transition point, all cities show fluctuations on $Y_i(t)$ indistinguishable from uncorrelated Gaussian noise. These two scaling regimes indicate that the network looks different in respect to the correlations of node ethnicity distribution when observed at local and global scale.

At small values of ε the walker is exploring the network for relative short time intervals (local scale) and it observes correlated fluctuations in the node population entropy, as indicated by the green fit line in Fig. 9. Conversely, at large values (blue fit line) the network appears uncorrelated ($\alpha \approx 1/2$), we denote the slope of this segment α_{tail}. The transition point marked by the vertical dashed line separating the two regimes (cut-off) corresponds to the typical walk length ε above which local heterogeneities and correlations in the values of $Y_i(t)$ are less significant. Therefore, for any length up to the transition point, the walker observes correlations on the fluctuations of φ, suggesting that there is no need to move too far across G to find the spatial patterns governing the population distribution.

Notably, for London, the coverage of neighbourhoods visited by W corresponds to an average area of 778, 779 and 796 Km^2 for Wards, LSOA and OA respectively. This area correspond to the walk length at the transition point, where London has a total area of 1572 Km^2. The distances were obtained by splinting $Y_i(t)$ on non overlapping segments, computing the total area covered at each segment and calculating the average over all segments. Detailed values for all UK cities analysed here are reported in Table 1. The mean covered area and the distance travelled within a city are relatively stable for all systems, indicating that the walker can extract the information about the correlations among the nodes of G no matter which spatial scale is used to delineate the territory.

Supplementary Table 1. Table showing the results of the DFA for the main metropolitan areas in the UK at at three distinct spatial scales. For all cities, the walks were simulated with lengths (number of steps) 1e+6, 50e+6 and 300e+6 for Wards, LSOA and OA respectively. Due to the larger size of the system, walks in London were defined with lengths 2e+6, 100e+6 and 900e+6 for wards, LSOA and OA respectively. The area covered is reported in Km^2 while the distance travelled in Km, and the corresponding standard deviation is indicated by STD.

			Slope		Cut-off		Area		Distance	
Met. area	Scale	Length	α	α_{tail}	ε	$F(\varepsilon)$	Mean	STD	Mean	STD
Bristol	Wards	4e+6	1.05	0.54	1.92	0.44	459.91	162.99	42.22	12.39
Bristol	LSOA	50e+6	1.13	0.54	2.67	1.21	465.05	183.05	41.50	12.30
Bristol	OA	300e+6	1.11	0.52	3.61	2.09	527.35	168.57	46.44	11.88
Cardiff	Wards	4e+6	1.01	0.50	2.67	0.98	1469.95	316.39	62.79	9.34
Cardiff	LSOA	50e+6	1.09	0.51	3.42	1.84	1952.08	385.57	69.17	7.89
Cardiff	OA	300e+6	1.09	0.52	3.99	2.38	1598.51	366.20	65.51	8.93
Liverpool	Wards	2e+6	1.00	0.55	2.11	0.46	304.57	77.61	31.35	6.52
Liverpool	LSOA	50e+6	1.10	0.52	2.86	1.27	224.37	59.37	28.49	6.37
Liverpool	OA	300e+6	1.08	0.51	3.80	2.12	318.34	72.56	33.72	6.46
London	Wards	6e+6	1.03	0.52	3.05	1.57	778.55	131.84	47.13	6.78
London	LSOA	100e+6	1.07	0.53	3.99	2.49	779.48	115.31	48.41	6.47
London	OA	900e+6	1.06	0.52	4.74	3.19	796.28	111.35	49.49	6.16
Manchester	Wards	4e+6	0.99	0.55	2.29	0.84	421.75	89.02	37.08	7.04
Manchester	LSOA	50e+6	1.08	0.55	3.24	1.77	427.80	86.87	37.85	6.83
Manchester	OA	300e+6	1.09	0.55	3.99	2.49	443.46	85.54	39.37	6.91
Northeast	Wards	2e+6	0.96	0.53	2.29	0.58	2270.74	1508.25	74.79	25.81
Northeast	LSOA	50e+6	1.07	0.52	2.86	1.20	1636.09	1522.99	63.96	31.42
Northeast	OA	300e+6	1.05	0.53	3.80	2.04	2254.10	1591.52	78.79	31.40
Sheffield	Wards	2e+6	0.86	0.50	2.67	0.98	1396.21	123.98	61.71	3.03
Sheffield	LSOA	50e+6	1.02	0.53	3.24	1.66	939.55	190.49	53.34	8.52
Sheffield	OA	300e+6	1.05	0.52	3.99	2.36	914.46	186.18	53.75	8.49
WMidlands	Wards	4e+6	0.99	0.53	1.92	0.56	200.85	66.02	25.41	6.65
WMidlands	LSOA	50e+6	1.11	0.53	3.05	1.68	197.82	52.11	26.89	6.51
WMidlands	OA	300e+6	1.10	0.53	3.99	2.53	282.34	63.33	32.50	7.31
WYorkshire	Wards	4e+6	0.85	0.51	2.29	0.71	1088.52	211.34	52.66	6.90
WYorkshire	LSOA	50e+6	1.07	0.53	2.86	1.40	428.86	137.81	38.28	8.39
WYorkshire	OA	300e+6	1.08	0.53	3.61	2.10	442.01	132.49	39.99	8.34

Supplementary Table 2. Properties of the metropolitan areas in the US and UK considered in this work. Nodes and links correspond to the spatially-embedded graph constructed from the neighbourhoods adjacency at the corresponding spatial scale of each country. Population data according to Census 2011 for the UK AND Census 2010 for US.

Met. area	Scale	Population	Classes	Nodes	Links	$\Delta\varrho$	$\Delta\sigma$	$\Delta\mu$
Atlanta	Census	5,618,431	60	1019	3172	311.23	15.68	1036.11
Boston	Census	7,558,009	63	1684	5130	667.81	23.35	4644.68
Chicago	Census	9,686,021	60	2273	7504	640.18	11.88	1317.09
Dallas	Census	6,726,779	60	1394	4466	479.62	11.03	1485.44
Houston	Census	6,045,555	62	1096	3603	441.93	13.18	1323.22
Los Angeles	Census	17,872,910	64	3923	12704	639.40	23.52	833.26
New York	Census	22,085,649	63	5277	16669	743.40	12.43	1666.32
Philadelphia	Census	6,533,683	60	1602	4940	452.69	11.23	840.99
San Francisco	Census	7,468,390	63	1651	5256	351.52	11.11	552.33
Washington	Census	8,572,971	63	2082	6600	613.15	10.57	1054.84
Bristol	Wards	1,069,583	227	143	402	228.49	16.84	646.96
Cardiff	Wards	1,480,251	222	287	801	612.37	17.15	2590.53
Liverpool	Wards	1,506,935	225	132	350	244.53	11.21	775.83
London	Wards	8,173,941	250	632	1859	49.03	21.08	268.49
Manchester	Wards	2,682,528	246	215	603	184.06	13.65	533.56
North East	Wards	1,934,095	225	241	669	308.14	20.02	985.77
Sheffield	Wards	1,343,601	226	91	245	177.64	13.62	478.73
West Midlands	Wards	2,736,460	249	163	436	157.02	14.42	357.70
West Yorkshire	Wards	2,226,058	239	124	345	136.36	13.84	194.42

Supplementary Table 3. Two-sided Spearman correlations of socio-economic variables for US metropolitan areas with CCT quantities and other segregation measures. Here we report the actual p-values (in brackets) of the symbolic annotation in Table 1 in the main manuscript. Values were obtained by using the *scipy.stats.spearmanr* python module.

	Diffusion				Moran I		Sp. Gini		Distance decay		Spatial dissimilarity		
Soc.-Econ. variables	$\Delta\mu$	$\Delta\varrho$	$\Delta\sigma$	$\tilde{\sigma}_{\langle\Gamma\rangle}$	$MI_{e[\Gamma]}$	$MI_{\langle\Gamma\rangle}$	$SG_{e[\Gamma]}$	$SG_{\langle\Gamma\rangle}$	$DDE_{\langle\Gamma\rangle}$	$DDI_{\langle\Gamma\rangle}$	$SD_{\langle\Gamma\rangle}$	$PSD_{\langle\Gamma\rangle}$	$BSD_{\langle\Gamma\rangle}$
Employment													
Employed	0.02 (0.676)	0.71 (0.002)	0.00 (0.855)	0.33 (0.082)	0.35 (0.074)	0.40 (0.048)	0.09 (0.405)	0.11 (0.347)	0.01 (0.751)	0.35 (0.074)	0.16 (0.260)	0.16 (0.260)	0.16 (0.260)
Unemployed	0.01 (0.803)	0.44 (0.038)	0.05 (0.533)	0.27 (0.128)	0.30 (0.098)	0.73 (0.002)	0.07 (0.446)	0.05 (0.533)	0.01 (0.829)	0.23 (0.162)	0.06 (0.489)	0.06 (0.489)	0.06 (0.489)
Commuting													
Car/truck/van (alone)	0.02 (0.676)	0.71 (0.002)	0.01 (0.777)	0.35 (0.074)	0.33 (0.082)	0.28 (0.117)	0.09 (0.405)	0.10 (0.385)	0.03 (0.651)	0.35 (0.074)	0.15 (0.276)	0.15 (0.276)	0.15 (0.276)
Public transportation	0.00 (0.934)	0.42 (0.043)	0.00 (0.987)	0.21 (0.187)	0.29 (0.108)	0.42 (0.043)	0.16 (0.260)	0.08 (0.425)	0.00 (0.881)	0.16 (0.260)	0.10 (0.365)	0.10 (0.365)	0.10 (0.365)
Walked	0.04 (0.603)	0.73 (0.002)	0.09 (0.405)	0.45 (0.033)	0.37 (0.060)	0.59 (0.009)	0.16 (0.260)	0.16 (0.260)	0.03 (0.651)	0.52 (0.019)	0.17 (0.229)	0.17 (0.229)	0.17 (0.229)
Other means	0.00 (0.960)	0.32 (0.090)	0.02 (0.701)	0.05 (0.533)	0.33 (0.082)	0.40 (0.048)	0.01 (0.777)	0.01 (0.803)	0.09 (0.405)	0.33 (0.082)	0.00 (0.855)	0.00 (0.855)	0.00 (0.855)
Worked at home	0.02 (0.676)	0.25 (0.138)	0.01 (0.829)	0.06 (0.489)	0.42 (0.043)	0.52 (0.019)	0.00 (0.960)	0.01 (0.803)	0.00 (0.881)	0.29 (0.108)	0.00 (0.881)	0.00 (0.881)	0.00 (0.881)
Occupation													
Management/science/arts	0.00 (0.907)	0.61 (0.008)	0.00 (0.881)	0.37 (0.060)	0.39 (0.054)	0.45 (0.033)	0.14 (0.293)	0.06 (0.511)	0.00 (0.934)	0.39 (0.054)	0.09 (0.405)	0.09 (0.405)	0.09 (0.405)
Service	0.01 (0.803)	0.69 (0.003)	0.01 (0.803)	0.42 (0.043)	0.32 (0.090)	0.45 (0.033)	0.13 (0.310)	0.14 (0.293)	0.02 (0.726)	0.36 (0.067)	0.16 (0.244)	0.16 (0.244)	0.16 (0.244)
Sales and office	0.04 (0.580)	0.73 (0.002)	0.00 (0.855)	0.33 (0.082)	0.32 (0.090)	0.36 (0.067)	0.13 (0.310)	0.10 (0.365)	0.00 (0.934)	0.25 (0.138)	0.17 (0.229)	0.17 (0.229)	0.17 (0.229)
Prod./transp./material	0.12 (0.328)	0.44 (0.038)	0.25 (0.138)	0.28 (0.117)	0.00 (0.987)	0.13 (0.310)	0.02 (0.726)	0.35 (0.074)	0.00 (0.934)	0.04 (0.603)	0.28 (0.117)	0.28 (0.117)	0.28 (0.117)
Occupation Industry													
Retail trade	0.03 (0.627)	0.73 (0.002)	0.03 (0.627)	0.33 (0.082)	0.39 (0.054)	0.56 (0.013)	0.14 (0.293)	0.10 (0.385)	0.00 (0.960)	0.30 (0.098)	0.16 (0.244)	0.16 (0.244)	0.16 (0.244)
Finan./real estate/leasing	0.07 (0.467)	0.79 (0.001)	0.03 (0.651)	0.36 (0.067)	0.33 (0.082)	0.45 (0.033)	0.16 (0.260)	0.13 (0.310)	0.00 (0.907)	0.27 (0.128)	0.22 (0.174)	0.22 (0.174)	0.22 (0.174)
Profe./scient./manag./adm.	0.00 (0.855)	0.40 (0.048)	0.00 (0.855)	0.19 (0.214)	0.39 (0.054)	0.42 (0.043)	0.01 (0.777)	0.00 (0.907)	0.00 (0.855)	0.39 (0.054)	0.01 (0.803)	0.01 (0.803)	0.01 (0.803)
Edu./health/social care	0.05 (0.533)	0.82 (0.000)	0.06 (0.489)	0.61 (0.008)	0.30 (0.098)	0.56 (0.013)	0.24 (0.150)	0.21 (0.187)	0.00 (0.907)	0.45 (0.033)	0.25 (0.138)	0.25 (0.138)	0.25 (0.138)
Arts/entert./accom./food	0.00 (0.987)	0.54 (0.016)	0.00 (0.987)	0.24 (0.150)	0.33 (0.082)	0.44 (0.038)	0.05 (0.533)	0.04 (0.556)	0.00 (0.855)	0.25 (0.138)	0.07 (0.446)	0.07 (0.446)	0.07 (0.446)
Income (USD)													
< $10,000	0.10 (0.385)	0.77 (0.001)	0.12 (0.328)	0.69 (0.003)	0.08 (0.425)	0.32 (0.090)	0.37 (0.060)	0.39 (0.054)	0.03 (0.651)	0.15 (0.276)	0.42 (0.043)	0.42 (0.043)	0.42 (0.043)
$10,000-$14,999	0.02 (0.676)	0.50 (0.022)	0.19 (0.214)	0.50 (0.022)	0.03 (0.651)	0.59 (0.009)	0.08 (0.425)	0.35 (0.074)	0.00 (0.960)	0.11 (0.347)	0.29 (0.108)	0.29 (0.108)	0.29 (0.108)
$15,000-$24,999	0.14 (0.293)	0.59 (0.009)	0.20 (0.200)	0.45 (0.033)	0.01 (0.829)	0.33 (0.082)	0.07 (0.467)	0.45 (0.033)	0.00 (0.907)	0.07 (0.446)	0.40 (0.048)	0.40 (0.048)	0.40 (0.048)
$25,000-$34,999	0.20 (0.200)	0.67 (0.004)	0.10 (0.385)	0.50 (0.022)	0.00 (0.907)	0.14 (0.293)	0.10 (0.365)	0.50 (0.022)	0.01 (0.751)	0.06 (0.489)	0.47 (0.029)	0.47 (0.029)	0.47 (0.029)
$50,000-$74,999	0.05 (0.533)	0.77 (0.001)	0.01 (0.829)	0.45 (0.033)	0.24 (0.150)	0.32 (0.090)	0.20 (0.200)	0.16 (0.244)	0.01 (0.829)	0.23 (0.162)	0.24 (0.150)	0.24 (0.150)	0.24 (0.150)
$75,000-$99,999	0.00 (0.881)	0.65 (0.005)	0.01 (0.777)	0.39 (0.054)	0.35 (0.074)	0.50 (0.022)	0.16 (0.260)	0.10 (0.385)	0.00 (0.907)	0.30 (0.098)	0.14 (0.293)	0.14 (0.293)	0.14 (0.293)
$100,000-$149,999	0.01 (0.829)	0.65 (0.005)	0.00 (0.907)	0.40 (0.048)	0.36 (0.067)	0.40 (0.048)	0.11 (0.347)	0.09 (0.405)	0.01 (0.751)	0.45 (0.033)	0.11 (0.347)	0.11 (0.347)	0.11 (0.347)
> $200,000	0.01 (0.803)	0.42 (0.043)	0.00 (0.881)	0.28 (0.117)	0.33 (0.082)	0.42 (0.043)	0.02 (0.726)	0.02 (0.701)	0.03 (0.651)	0.47 (0.029)	0.02 (0.701)	0.02 (0.701)	0.02 (0.701)
Supplementary Security													
Social Security	0.01 (0.751)	0.71 (0.002)	0.06 (0.489)	0.65 (0.005)	0.19 (0.214)	0.59 (0.009)	0.28 (0.117)	0.24 (0.150)	0.00 (0.907)	0.24 (0.150)	0.28 (0.117)	0.28 (0.117)	0.28 (0.117)
Cash public assist.	0.00 (0.881)	0.47 (0.029)	0.04 (0.580)	0.73 (0.002)	0.06 (0.511)	0.59 (0.009)	0.17 (0.229)	0.23 (0.162)	0.00 (0.855)	0.19 (0.214)	0.20 (0.200)	0.20 (0.200)	0.20 (0.200)
Food Stamp/SNAP 12m	0.12 (0.328)	0.75 (0.001)	0.13 (0.310)	0.67 (0.004)	0.04 (0.556)	0.30 (0.098)	0.32 (0.090)	0.42 (0.043)	0.04 (0.580)	0.10 (0.365)	0.45 (0.033)	0.45 (0.033)	0.45 (0.033)

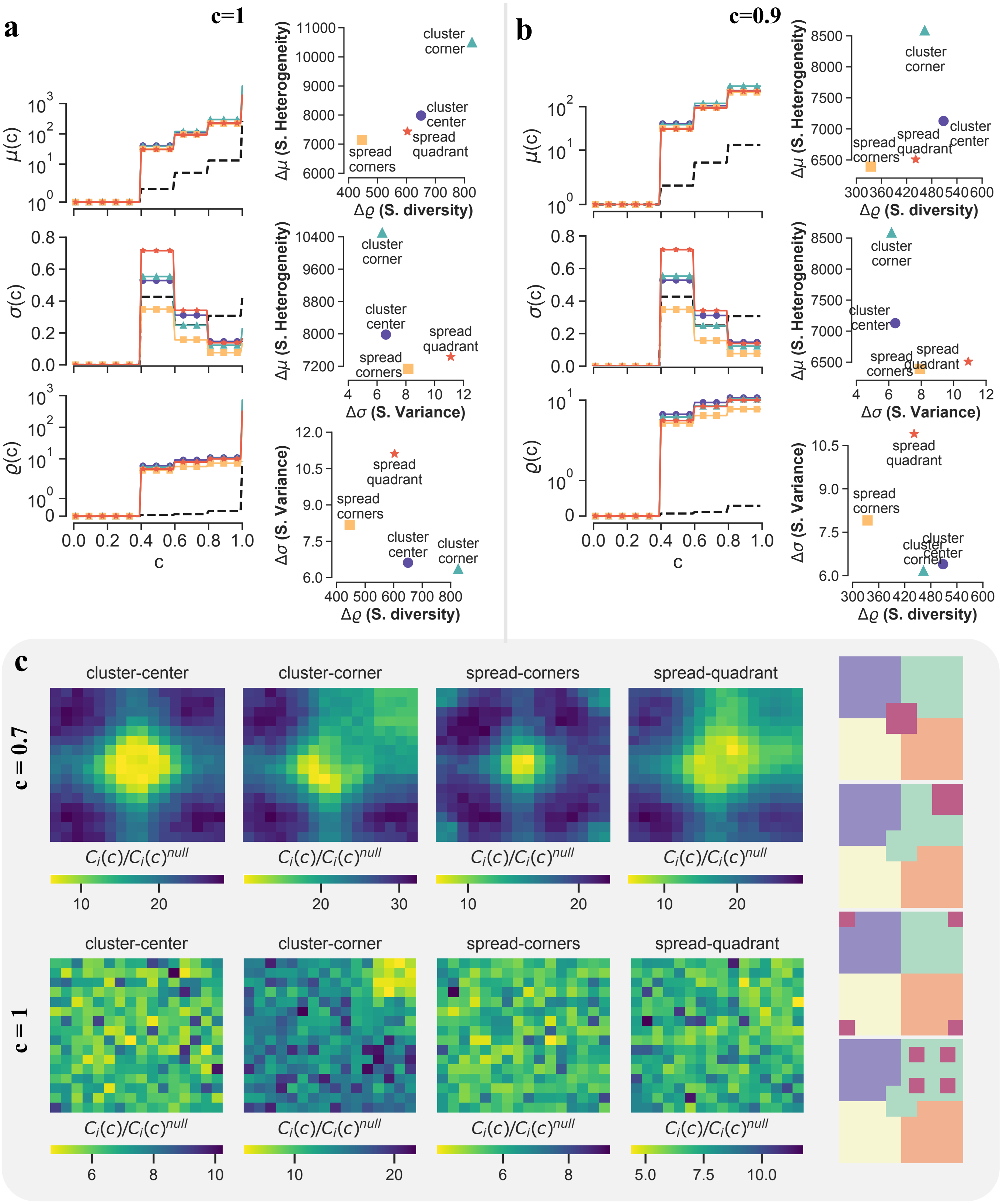

Supplementary Figure 1. Effect of a minority class on class coverage times distributions. (**a**) CCT Distributions for the 2-D lattice with five classes as presented in Fig. 2 in the main text. (**b**) As in the left panel but the values for $c = 1$ are removed to isolate the effect of the less abundant class. (**c**) Heatmaps reporting the normalised coverage times $C_i(c)/C_i(c)^{null}$ for $c = 0.7$ and $c = 1$. The dependency on the starting node is less pronounced on the later while the range of values in the colourbar is somehow preserved.

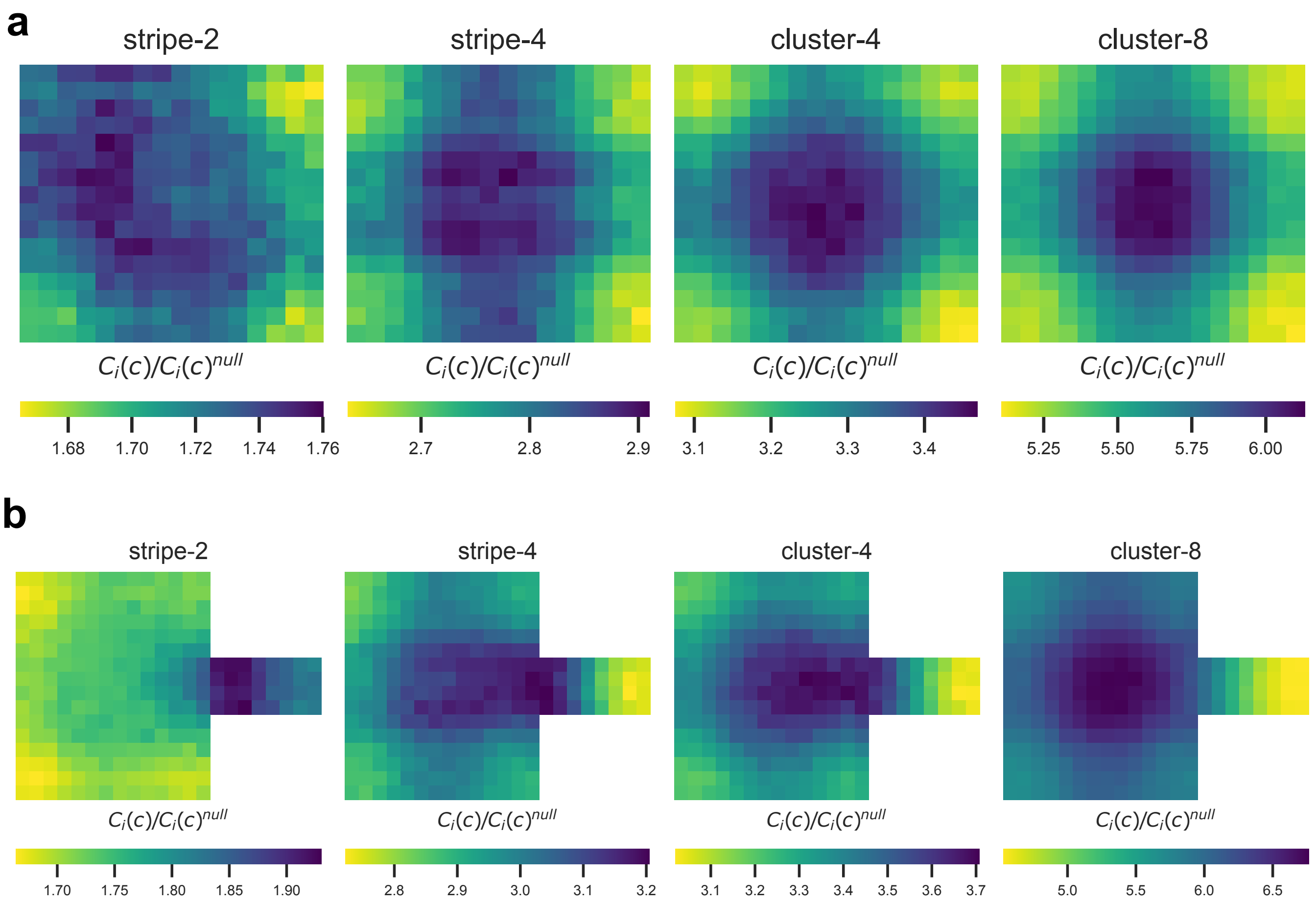

Supplementary Figure 2. Heatmaps reporting the normalised coverage times $C_i(c)/C_i(c)^{null}$ for $c = 0.7$ of the synthetic colourings of lattices in Fig.4 of the main text. **(a)** The population is divided in 32 classes placed randomly with 4 different setups on on a 2-D lattice. **(b)** Similar to **(a)** but the domain has a lateral appendix.

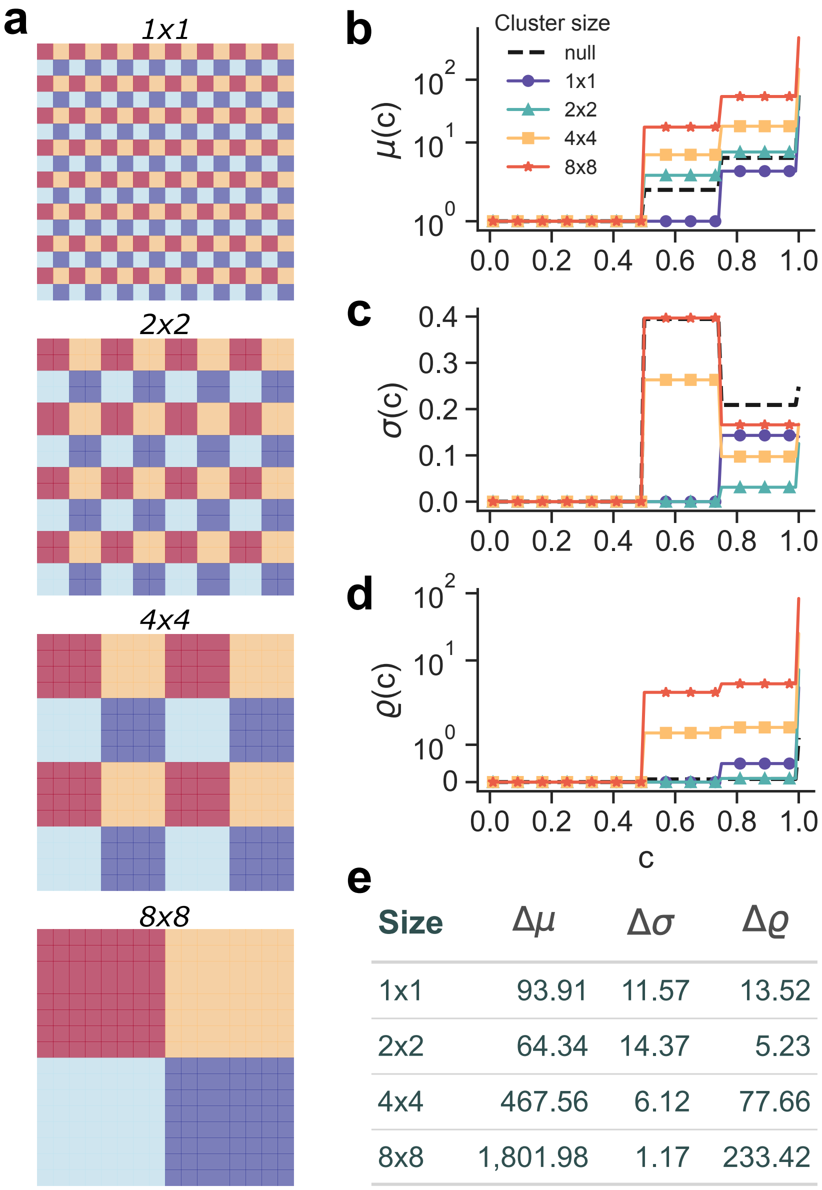

Size	Δμ	Δσ	Δϱ
1x1	93.91	11.57	13.52
2x2	64.34	14.37	5.23
4x4	467.56	6.12	77.66
8x8	1,801.98	1.17	233.42

Supplementary Figure 3. Diffusion-based segregation measures on square lattices with checkboard-like arrangements of four classes, for different sizes of clusters. Notice that all the three quantities are sensitive to the size of the homogeneous clusters forming the pattern.

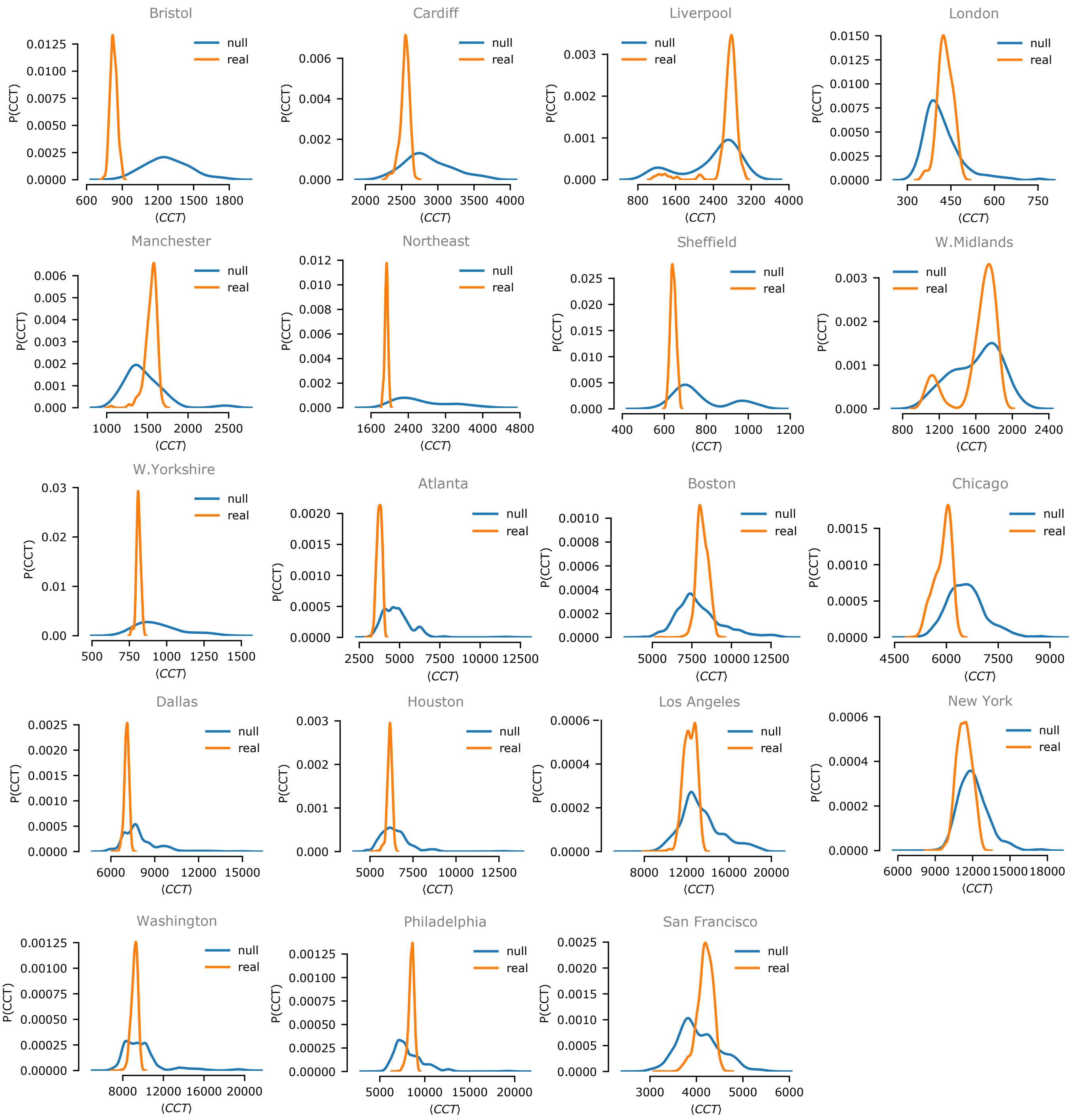

Supplementary Figure 4. Distribution of the Class Coverage Time over the wards of metropolitan areas in the UK and UK, where larger values of CCT correspond to more segregated areas. The estimates for CCT are obtained using 1.000 distinct trajectories from each node. The observed distributions are qualitatively and quantitatively distinct from the corresponding distribution in a null-model where the profiles of classes is reshuffled uniformly at random (100 different realisations). The population is divided into up to $\Gamma = 250$ different classes for the UK Census data and up to $\Gamma = 64$ classes for the American Census Bureau data.

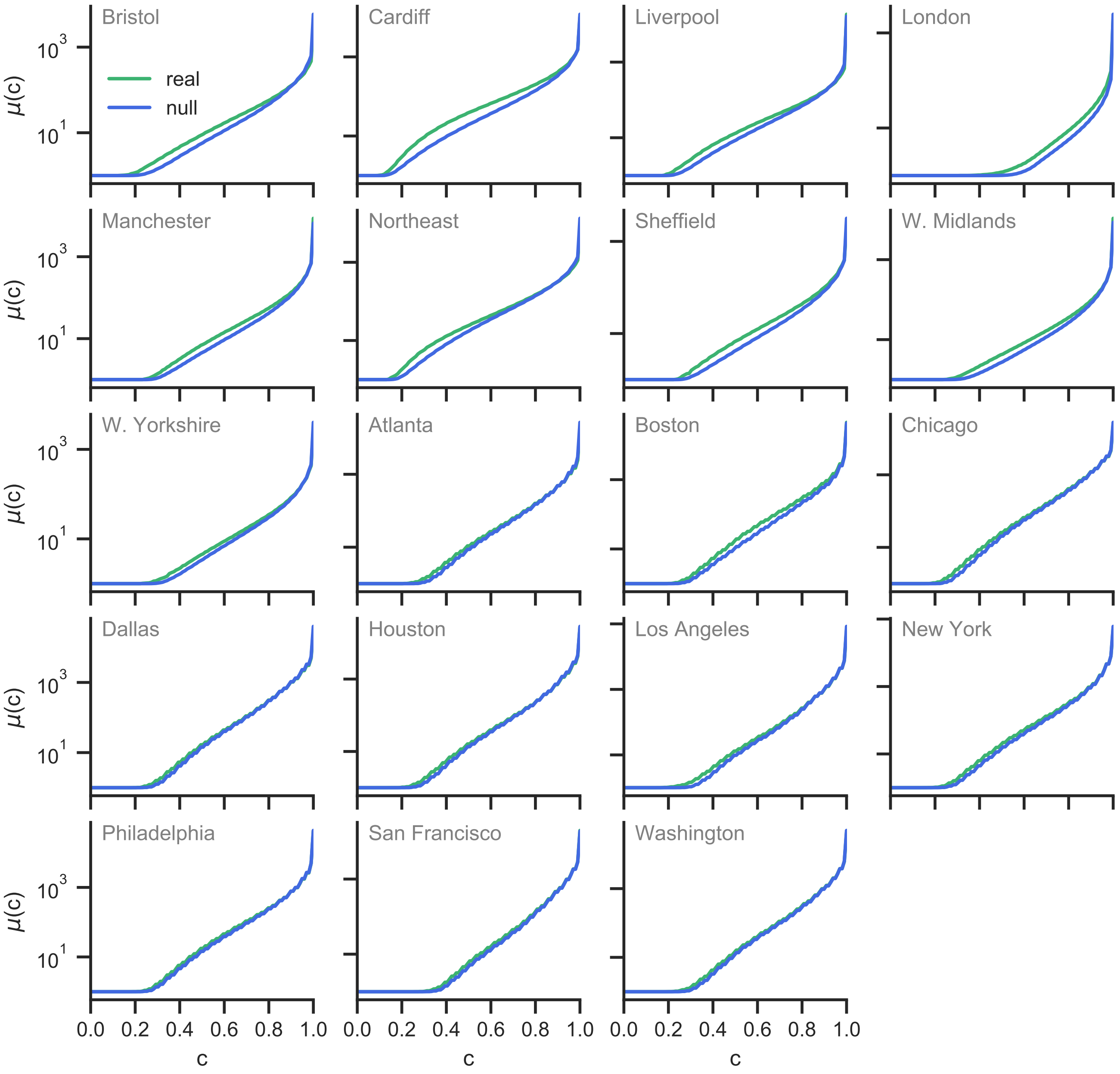

Supplementary Figure 5. Mean time $\mu(c)$ as a function of fractions c of classes in the real system and the corresponding null model for the metropolitan areas in the UK and US.

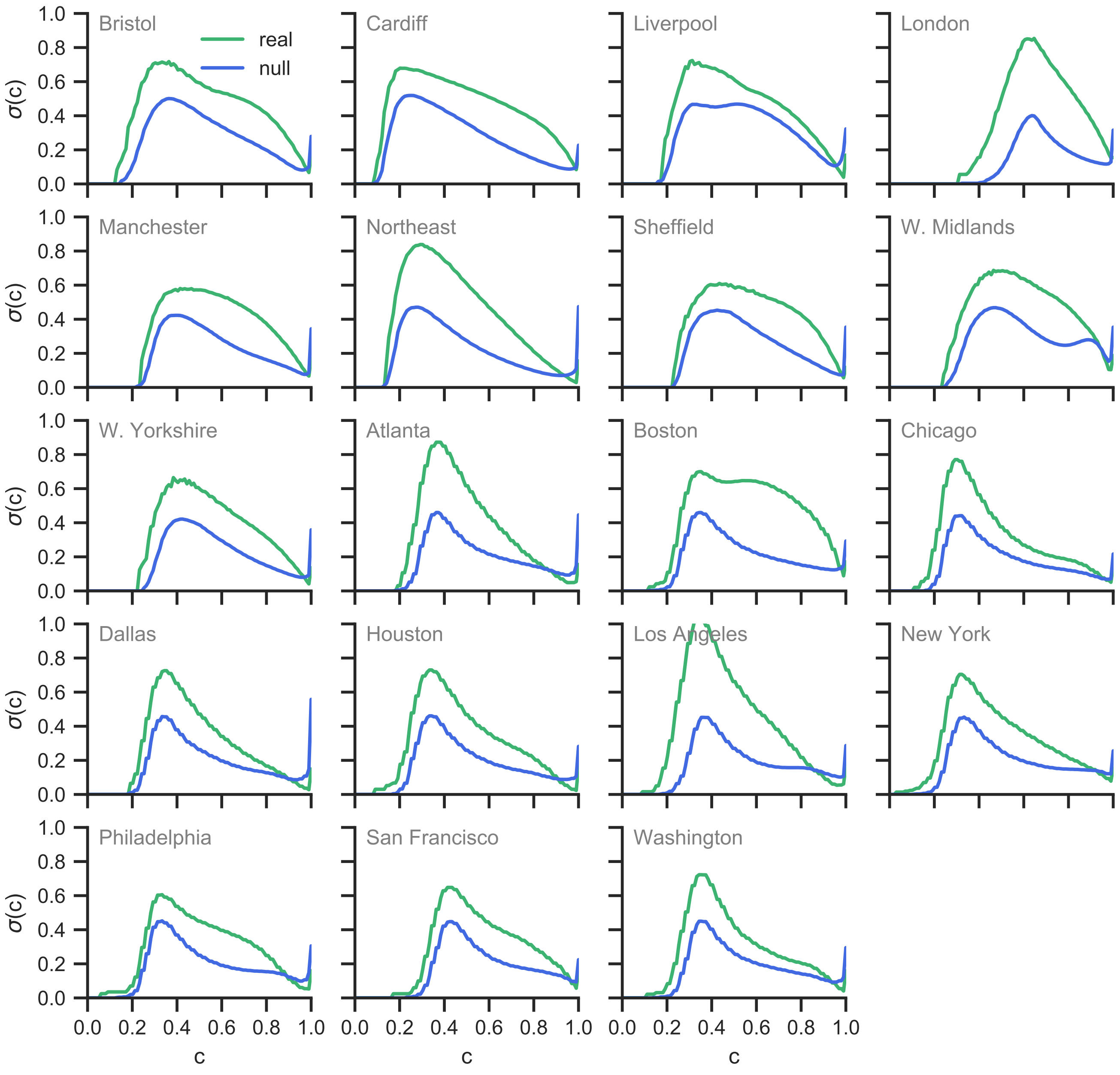

Supplementary Figure 6. Coefficient of variance $\sigma(c)$ as a function of fractions c of classes in the real system and the corresponding null model for the metropolitan areas in the UK and US. The values of $\sigma(c)$ are normalised by the respective mean $\mu(c)$. Values for $c = 1$ are kept for demonstration of the spurious effects. The flat line for small values of c is due to the number of classes observed at the initial node i being larger than the fraction c considered.

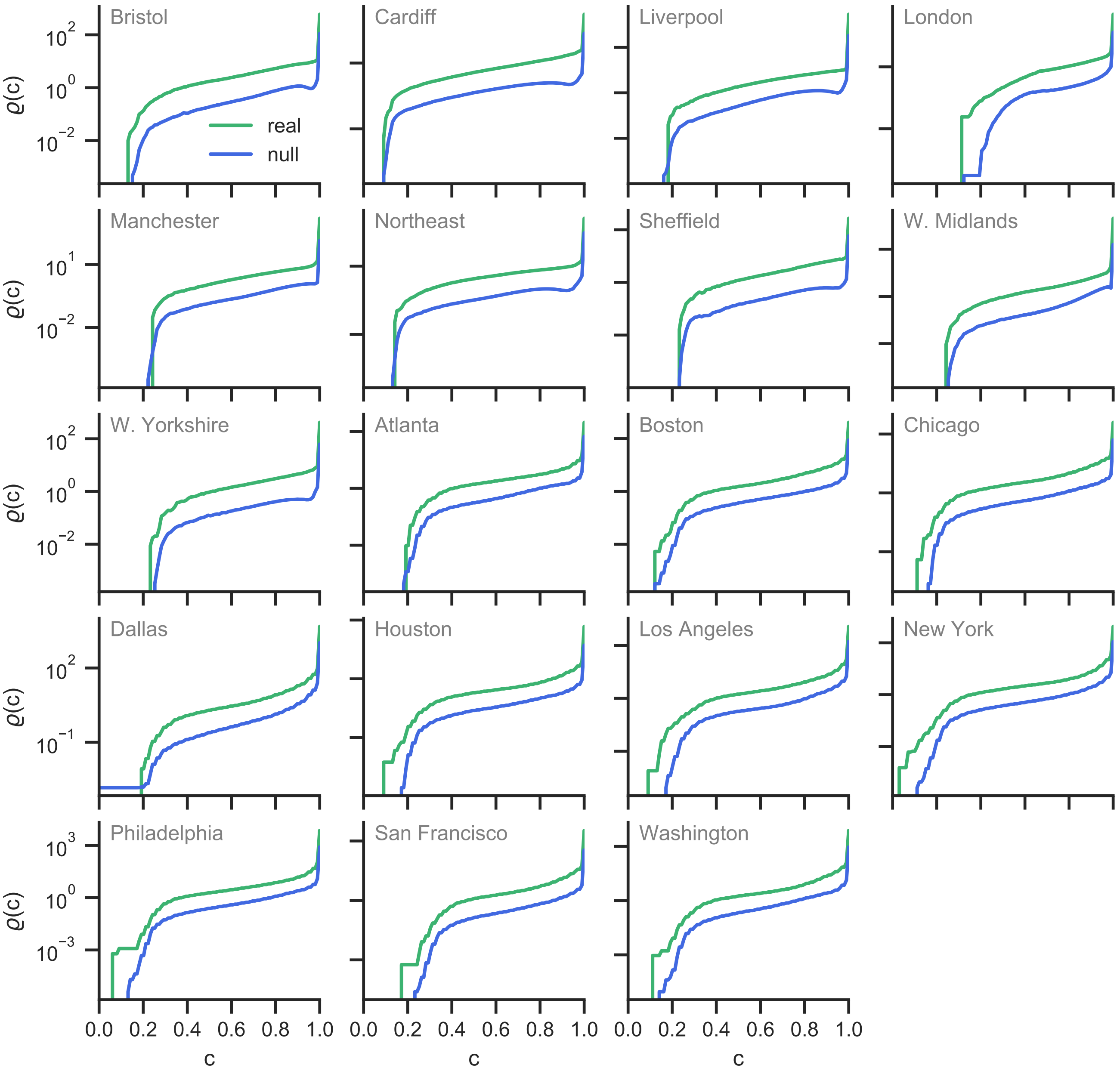

Supplementary Figure 7. Spatial diversity $\varrho(c)$ as a function of fractions c of classes in the real system and the corresponding null model for the metropolitan areas in the UK and US.

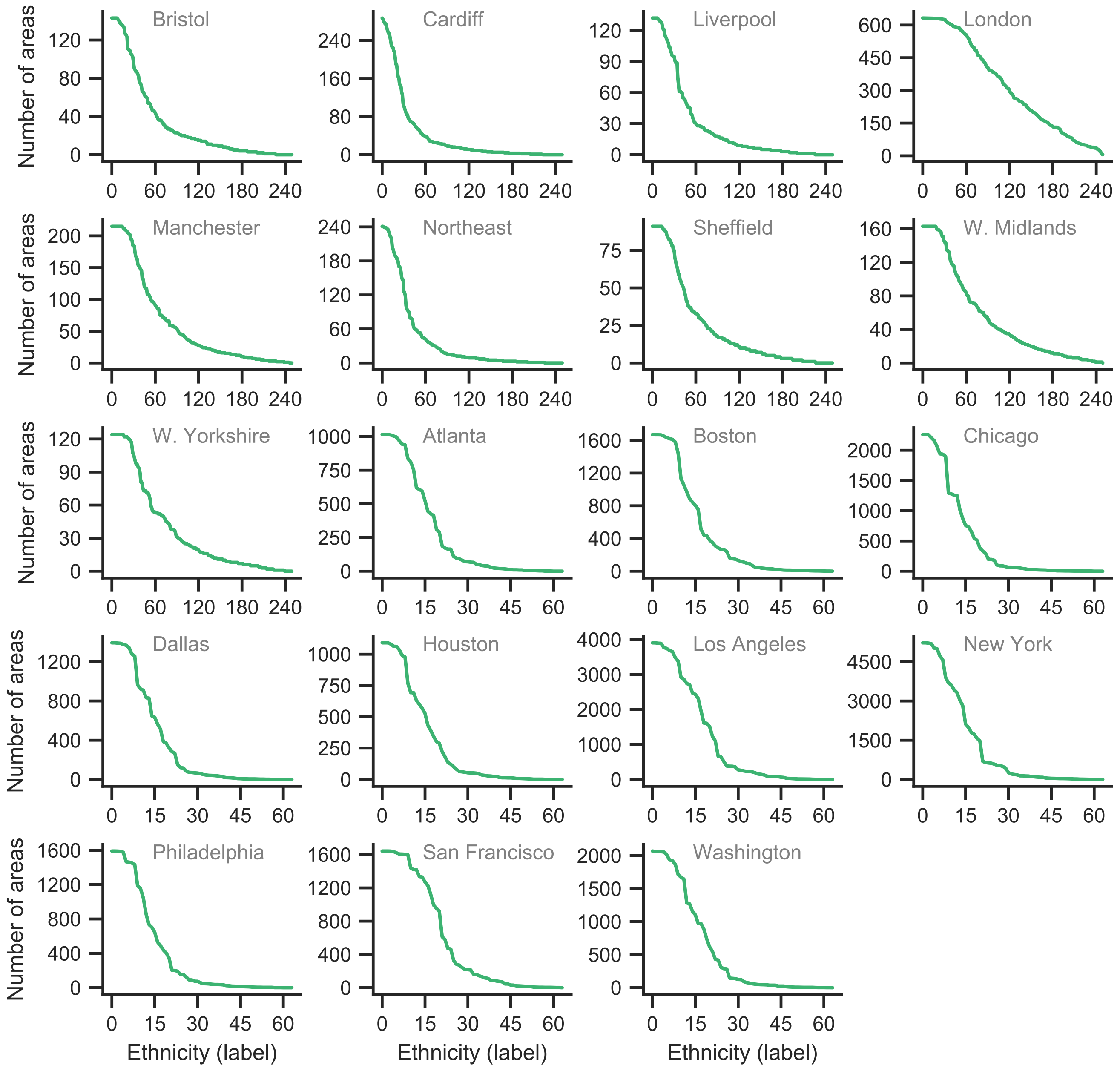

Supplementary Figure 8. Number of areal units at which an ethnicity can be found in the metropolitan area in the UK and US. The number labelling the ethnicity corresponds to the column sequence from the UK and US corresponding Census tables. Notably, the vertical axis varies substantially among cities, and some ethnic groups are present only at a small number of neighbourhoods, consequently affecting the CCT of the respective nodes.

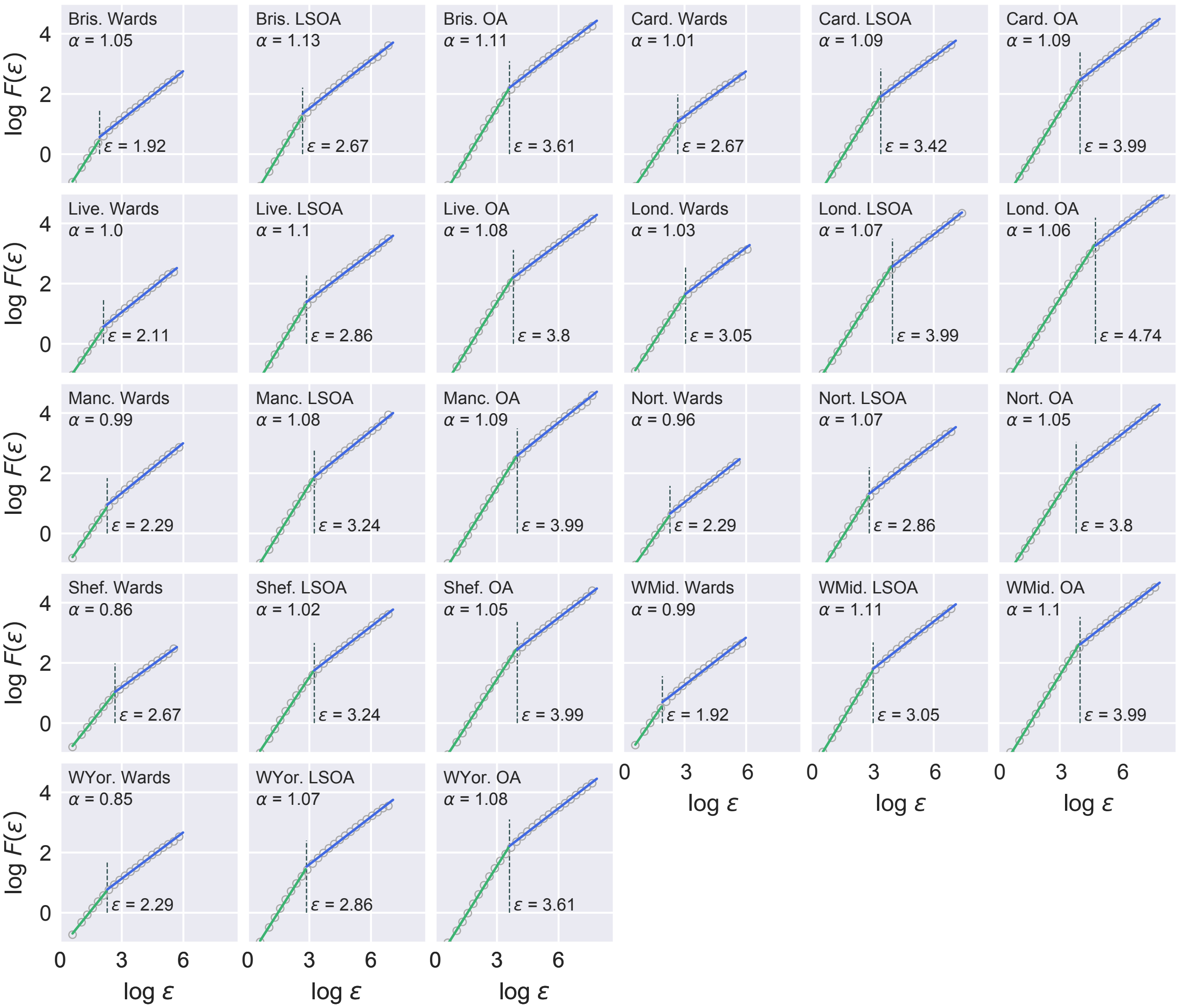

Supplementary Figure 9. Multifractal detrended fluctuation analysis of the UK cities at three distinct spatial scales. The DFA of the node population entropy reveals two distinct spatial scale regimes across the neighbourhoods of the metropolitan areas in the UK at three different granularities: Wards, Lower Layer Super Output Areas (LSOA) and Output Areas (OA). The value $F(\varepsilon)$ is plotted as a function of ε in log-log scale.

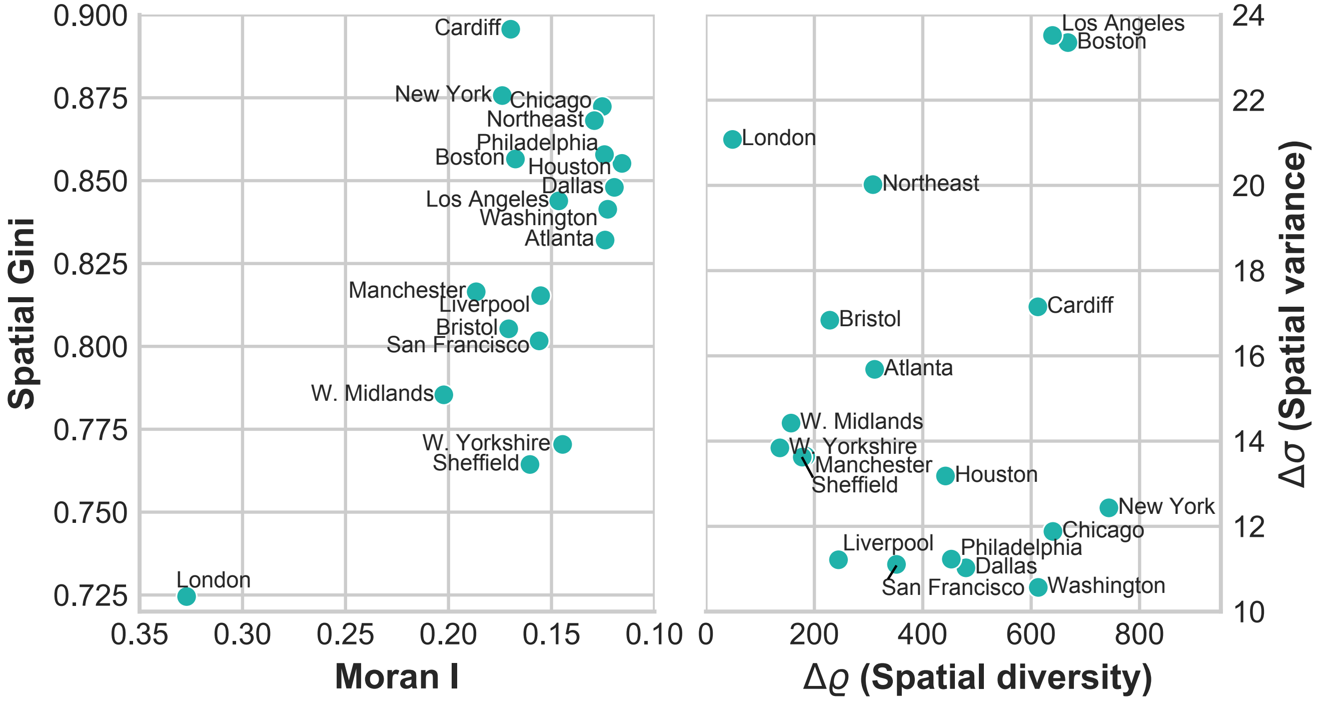

Supplementary Figure 10. Quantifying spatial ethnic segregation in urban systems. Alternative view for Fig. 4 in the main text where the results of the Spatial Gini coefficient and Moran I are obtained by calculating the indices for each class in the city and averaging over all classes.

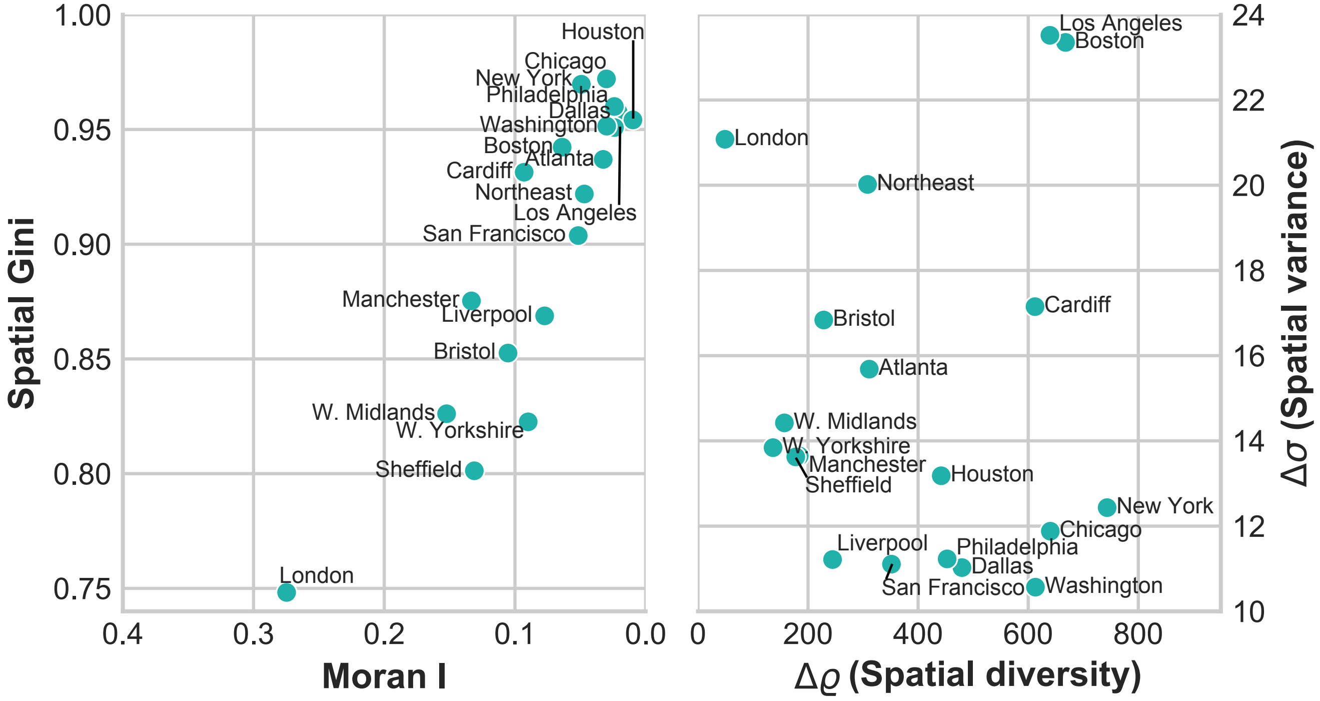

Supplementary Figure 11. Quantifying spatial ethnic segregation in urban systems. Alternative view for Fig. 4 in the main text where the results of the Spatial Gini coefficient and Moran I are obtained by calculating the indices for each class in the city and computing the median of all classes.

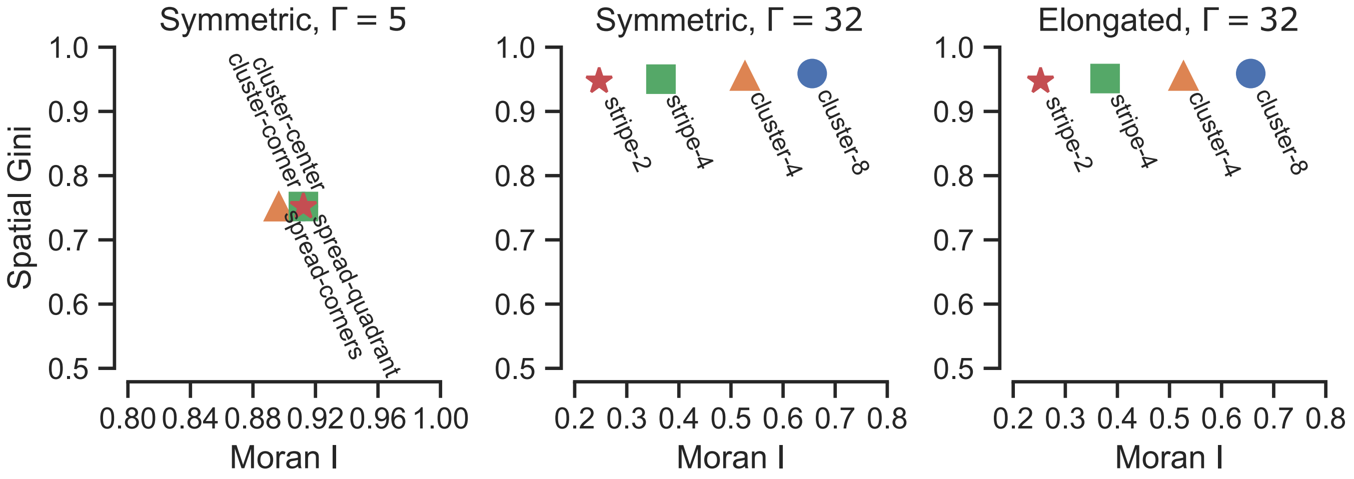

Supplementary Figure 12. Quantifying spatial ethnic segregation on synthetic systems. Spatial Gini coefficient and Moran I are reported for the synthetic systems illustrated in the main manuscript. Values are obtained by calculating the indices for each class in the system and computing the median of all classes.

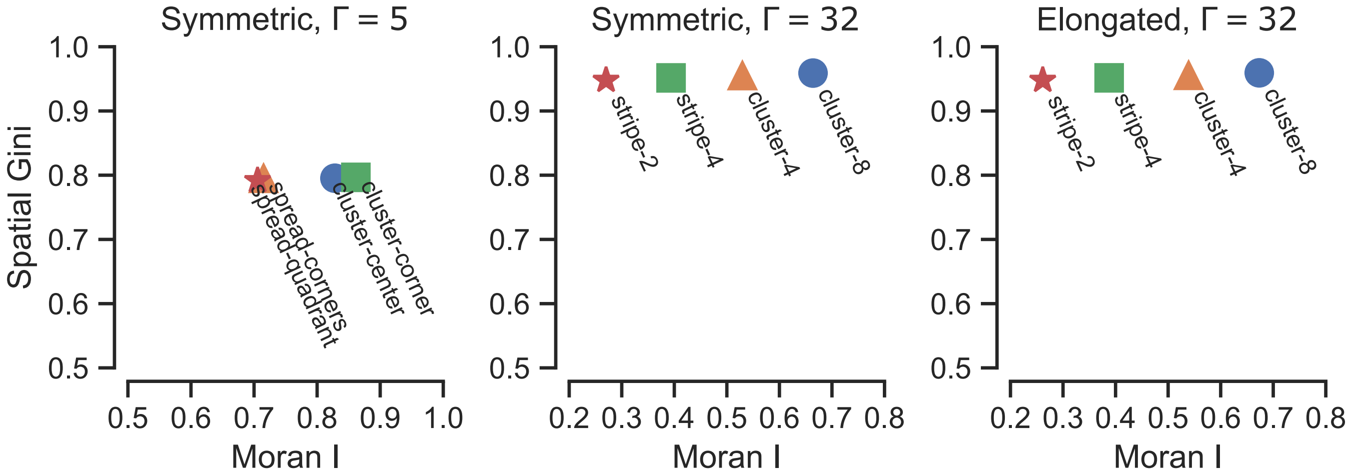

Supplementary Figure 13. Quantifying spatial ethnic segregation on synthetic systems. Spatial Gini coefficient and Moran I are reported for the synthetic systems illustrated in the main manuscript. Values are obtained by calculating the indices for each class in the system and computing the average of all classes.

[1] Kantelhardt, J.W., Zschiegner, S.A., Konscienly-Bunde, E., Havlin, S., Bunde, A. and Stanley, H.E. (2002) Multifractal detrended fluctuation analysis of nonstationary time series. Physica A: Statistical Mechanics and Its Applications, 316, 87-114. doi:10.1016/S0378-4371(02)01383-3
