# Supplementary material for: Quantifying ethnic segregation in cities through random walks
Source: Nat Commun. 2022 Oct 3;13:5809. doi: 10.1038/s41467-022-33344-3 (PMC9530170; doi:10.1038/s41467-022-33344-3)
Supplement: Supplementary file 2 — Reporting Summary [file 41467_2022_33344_MOESM2_ESM.pdf]

nature portfolio

Corresponding author(s): Vincenzo Nicosia

Last updated by author(s): Sep 13, 2022

Reporting Summary

Nature Portfolio wishes to improve the reproducibility of the work that we publish. This form provides structure for consistency and transparency in reporting. For further information on Nature Portfolio policies, see our Editorial Policies and the Editorial Policy Checklist.

Statistics

For all statistical analyses, confirm that the following items are present in the figure legend, table legend, main text, or Methods section.

n/a	Confirmed	
☐	☒	The exact sample size (*n*) for each experimental group/condition, given as a discrete number and unit of measurement
☐	☒	A statement on whether measurements were taken from distinct samples or whether the same sample was measured repeatedly
☐	☒	The statistical test(s) used AND whether they are one- or two-sided<br>*Only common tests should be described solely by name; describe more complex techniques in the Methods section.*
☐	☒	A description of all covariates tested
☐	☒	A description of any assumptions or corrections, such as tests of normality and adjustment for multiple comparisons
☐	☒	A full description of the statistical parameters including central tendency (e.g. means) or other basic estimates (e.g. regression coefficient) AND variation (e.g. standard deviation) or associated estimates of uncertainty (e.g. confidence intervals)
☒	☐	For null hypothesis testing, the test statistic (e.g. *F*, *t*, *r*) with confidence intervals, effect sizes, degrees of freedom and *P* value noted<br>*Give P values as exact values whenever suitable.*
☒	☐	For Bayesian analysis, information on the choice of priors and Markov chain Monte Carlo settings
☒	☐	For hierarchical and complex designs, identification of the appropriate level for tests and full reporting of outcomes
☒	☐	Estimates of effect sizes (e.g. Cohen's *d*, Pearson's *r*), indicating how they were calculated

Our web collection on statistics for biologists contains articles on many of the points above.

Software and code

Policy information about availability of computer code

Data collection	The ethnicity data that supports the findings of this study has been obtained from the UK and US census agencies, and is openly available. A cleaned-up version of the data, as used in the study, is available in the repository at: https://github.com/segregation-rw/ethnic-segregation-rw. The data generated by the random walk process on the graphs is available at https://doi.org/10.5281/zenodo.5521053
Data analysis	All the custom code use for the analysis is available in the repository at: https://github.com/segregation-rw/ethnic-segregation-rw

For manuscripts utilizing custom algorithms or software that are central to the research but not yet described in published literature, software must be made available to editors and reviewers. We strongly encourage code deposition in a community repository (e.g. GitHub). See the Nature Portfolio guidelines for submitting code & software for further information.

Data

Policy information about availability of data

All manuscripts must include a data availability statement. This statement should provide the following information, where applicable:

- Accession codes, unique identifiers, or web links for publicly available datasets
- A description of any restrictions on data availability
- For clinical datasets or third party data, please ensure that the statement adheres to our policy

The ethnicity data that supports the findings of this study section is openly available at the respective Census agencies at http://dx.doi.org/10.5257/census/aggregate-2011-1 and http://doi.org/10.18128/D050.V14.0. A cleaned version of the input files is available in the repository https://github.com/segregation-rw/ethnic-segregation-rw. The data generated by the random walk process for all experiments are available in the repository: https://doi.org/10.5281/zenodo.5521053

Human research participants

Policy information about studies involving human research participants and Sex and Gender in Research.

Reporting on sex and gender	N/A
Population characteristics	N/A
Recruitment	N/A
Ethics oversight	N/A

Note that full information on the approval of the study protocol must also be provided in the manuscript.

Field-specific reporting

Please select the one below that is the best fit for your research. If you are not sure, read the appropriate sections before making your selection.

☐ Life sciences ☐ Behavioural & social sciences ☒ Ecological, evolutionary & environmental sciences

For a reference copy of the document with all sections, see nature.com/documents/nr-reporting-summary-flat.pdf

Ecological, evolutionary & environmental sciences study design

All studies must disclose on these points even when the disclosure is negative.

Study description	The study proposes a set of measures for the quantification of spatial heterogeneity and segregation, and applies those measures to the analysis of ethnic segregation in urban areas.
Research sample	The ethnic data was selected on the basis of availability. We selected data about the largest metropolitan areas in the UK and in the US, since for those cities we also have information about social deprivation indices.
Sampling strategy	We selected the 10 largest metropolitan areas by population in the UK and in the US. The selection of the largest metropolitan areas was due to the possible presence of confounding factors and poor statistics in smaller urban areas, especially regarding the presence of a large variety of different ethnicities.
Data collection	The data was already existing, and consists of UK and US census data about the selected metropolitan areas, as well as the corresponding maps of census tracts, which were converted into graphs. In those graphs, each node corresponds to a census tract and two nodes are connected by an edge if the corresponding census tracts border each other.
Timing and spatial scale	The spatial scale is the corresponding administrative boundary definition as detailed in each census data set. For the UK data, census information is from the 2011 census aggregate data. Fof the US data set, census information comes from the American Community Survey 2011
Data exclusions	No data were excluded
Reproducibility	The results are easily reproducible using the open source software provided, which implements all the proposed measures.
Randomization	The only randomization procedure is the one employed to obtain null models of the spatial distributions of ethnic groups. Each null model realization was obtained by reassigning the vectors of census tracts distributions uniformly at random among nodes.
Blinding	Blinding not relevant. We did not make any specific assumption or selection of features or aspects. The data was used exactly as made available in the corresponding census data sets.

Did the study involve field work? ☐ Yes ☒ No

Reporting for specific materials, systems and methods

We require information from authors about some types of materials, experimental systems and methods used in many studies. Here, indicate whether each material, system or method listed is relevant to your study. If you are not sure if a list item applies to your research, read the appropriate section before selecting a response.

Materials & experimental systems

n/a	Involved in the study
☒	☐ Antibodies
☒	☐ Eukaryotic cell lines
☒	☐ Palaeontology and archaeology
☒	☐ Animals and other organisms
☒	☐ Clinical data
☒	☐ Dual use research of concern

Methods

n/a	Involved in the study
☒	☐ ChIP-seq
☒	☐ Flow cytometry
☒	☐ MRI-based neuroimaging
